# Diversity and extracellular enzymatic activity of culturable yeasts isolated from soil in a Brazilian Amazonian rainforest biome

**DOI:** 10.1007/s42770-026-01984-y

**Published:** 2026-06-01

**Authors:** Ana Raquel O. Santos, Fernanda L. C. Oliveira, Arthur N. Sander, Gisele F. L. Souza, Katharina O. Barros, Maxwel A. Abegg, Marc-André Lachance, Carlos Augusto Rosa

**Affiliations:** 1 Departamento de Microbiologia, ICB, Universidade Federal de Minas Gerais, C.P. 486, Belo Horizonte, MG 31270-901 Brazil; 2 Instituto de Ciências Exatas e Tecnologia, Universidade Federal do Amazonas, Itacoatiara, AM 69100-000 Brazil; 3 Department of Biology, University of Western Ontario, London, N6A 5B7 Canada

**Keywords:** Soil yeasts, Amazon rainforest, Diversity, Enzymes

## Abstract

The Amazon rainforest represents the largest and most biodiverse tropical rainforest in the world, comprising over half of the planet’s remaining rainforests. In this study, we investigated the yeast distribution in the soils in a Brazilian Amazonian rainforest biome. The collection sites included two upland forests, a black-water floodplain forest, and an Amazonian dark earth site. Yeasts were isolated using both enrichment and direct isolation techniques, with different incubation temperatures. We obtained a total of 598 yeast and yeast-like isolates belonging to 110 species. The highest yeast diversity was obtained using the enrichment isolation procedure. Species richness and Shannon diversity were higher in the upland forests than in the other sites. *Lipomyces* species were only recovered when a nitrogen-free medium was used. Among 314 isolates tested, 85.5% produced at least one extracellular enzyme capable of degrading proteins, esters, xylan, starch, lipids, pectin, cellulose, or tannins. About 45% of the yeasts also solubilized phosphate. Forty-five possible candidates for new species were identified, indicating that soil is a significant source of novel yeasts in Amazonian rainforest biomes.

## Introduction

Soils are considered reservoirs for yeasts. These microorganisms contribute to the mineralization of organic matter, phosphate solubilization, plant root protection against fungal pathogens, seed germination, enhancement of plant growth promoters, or serve as nutritional sources for other soil-dwellers [1–7]. Soil yeast communities are taxonomically diverse and possess adaptations that allow them to survive in a wide range of environmental conditions [3]. Soil moisture, pH, carbon, and nitrogen content have been suggested as factors affecting soil yeast diversity [1].

Yeasts are capable of producing enzymes such as cellulases, amylases, xylanases, lipases, proteases, and pectinases, which enhance soil fertility and promote plant growth. Moreover, the solubilization of micronutrients such as phosphorus and zinc, mediated by the secretion of organic acids and enzymes, further improves soil health, thereby supporting plant development [7]. Consequently, yeasts act as key mediators of nutrient turnover and trophic interactions in soil ecosystems, strengthening the functional link between microbial communities and plant roots.

Mestre et al. [8] studied soil yeast communities in different sites dominated by *Nothofagus pumilio* (two sites) and *N. antarctica* (two sites) in Northern Patagonian forests in Argentina. The authors found that each forest site exhibited a distinct species composition as a result of environmental characteristics such as dominant plant species, nutrient availability, and climate conditions. *Cryptococcus podzolicus* (= *Saitozyma podzolica*) was the most frequent species in nutrient-rich soils, *Trichosporon porosum* in cold mountain forest soils with low nutrient and water availability, and *Cr. phenolicus* in sites with low precipitation. Yurkov et al. [9], in a study conducted in Serra da Arrábida Natural Park (Portugal), reported that the structure of soil yeast communities reflects the environmental factors that influence aboveground phytocenoses, aboveground biomass, and plant projective cover.

Samarasinghe et al. [5], using a global collection of nearly 4,000 soil samples, assessed culturable soil yeast diversity in nine countries representing all continents except Antarctica. These authors obtained a total of 1,473 isolates and found that soil yeast populations were distinct in structure and composition between countries, with 73% of the discovered yeast species found in only one of the sampled countries. Mean annual precipitation was the most significant predictor of the soil yeast communities on a global scale, and air traffic volume was correlated with the number of shared species between countries. Spurley et al. [10] collected samples of natural substrates (soil, bark, leaves, fruit, fungus, plant matter, sand, flowers, lichen, insects, and feathers) across the continental USA and Alaska. Soil was the most heavily sampled substrate and was estimated to have the highest species richness. Two Basidiomycota (*Mrakia* spp. complex and *Trichosporon porosum*) and three Saccharomycotina yeast species (*Torulaspora delbrueckii*, *Cyberlindnera saturnus*, and *Saccharomyces paradoxus*) were significantly isolates from this substrate in the region studied. These works demonstrate that soil yeast communities have distinct species compositions across different biomes, with the occurrence of both endemic and cosmopolitan species.

Most studies on the diversity of soil yeast communities have been conducted in temperate climate biomes. In tropical ecosystems, only a few studies on the diversity of these yeast communities have been published [11–17]. Some of these studies focused on the search for opportunistic pathogenic yeasts or species of biotechnological interest [11, 17, 18].

In the Brazilian Amazon rainforest, only a few studies have analyzed soil yeast communities. Two major studies have investigated the diversity of soil yeasts in this region; however, these studies focused on pathogenic [11] and mycocinogenic [12] yeasts. These studies did not use DNA sequencing to confirm yeast identities; the isolates were identified based on physiological and morphological characteristics, making it likely that some species were misidentified.

Hoang and Kanemoto [19] reported that over half of the Earth’s remaining tropical rainforests are located in the Amazon Basin, where the deforestation rate has increased rapidly in recent years. About 60% of the Amazon rainforest coverage lies in Brazil [20]. Considering the great diversity of plants and animals in the region, a huge microbial diversity is also expected, as microorganisms colonize all natural plant cover of the soil and other above-ground substrates [21, 22]. Barros et al. [23] investigated the yeast biodiversity associated with rotting wood in a Brazilian Amazon rainforest biome and showed that 36% of the yeasts identified were candidates for novel species, including those belonging to the genera *Spathaspora*, *Scheffersomyces*, and *Sugiyamaella*, which have strains with potential biotechnological applications. These authors demonstrated that rotting wood collected from the Amazon rainforest was a tremendous source of diverse yeast communities.

To increase our knowledge of yeast biodiversity in the Brazilian Amazon rainforest, we investigated the diversity of soil yeast communities present in three different forest types found in an Amazon rainforest biome. The yeast species were obtained using direct and enrichment isolation techniques, as well as different incubation temperatures. A specific medium for isolating *Lipomyces* species was also used. Additionally, we determined the ability of the yeast isolates to solubilize phosphate and to produce hydrolytic enzymes that could contribute to the ecological roles of these microorganisms in soil and might be of biotechnological interest.

## Materials & methods

### Study sites and sampling

**Fig. 1:**
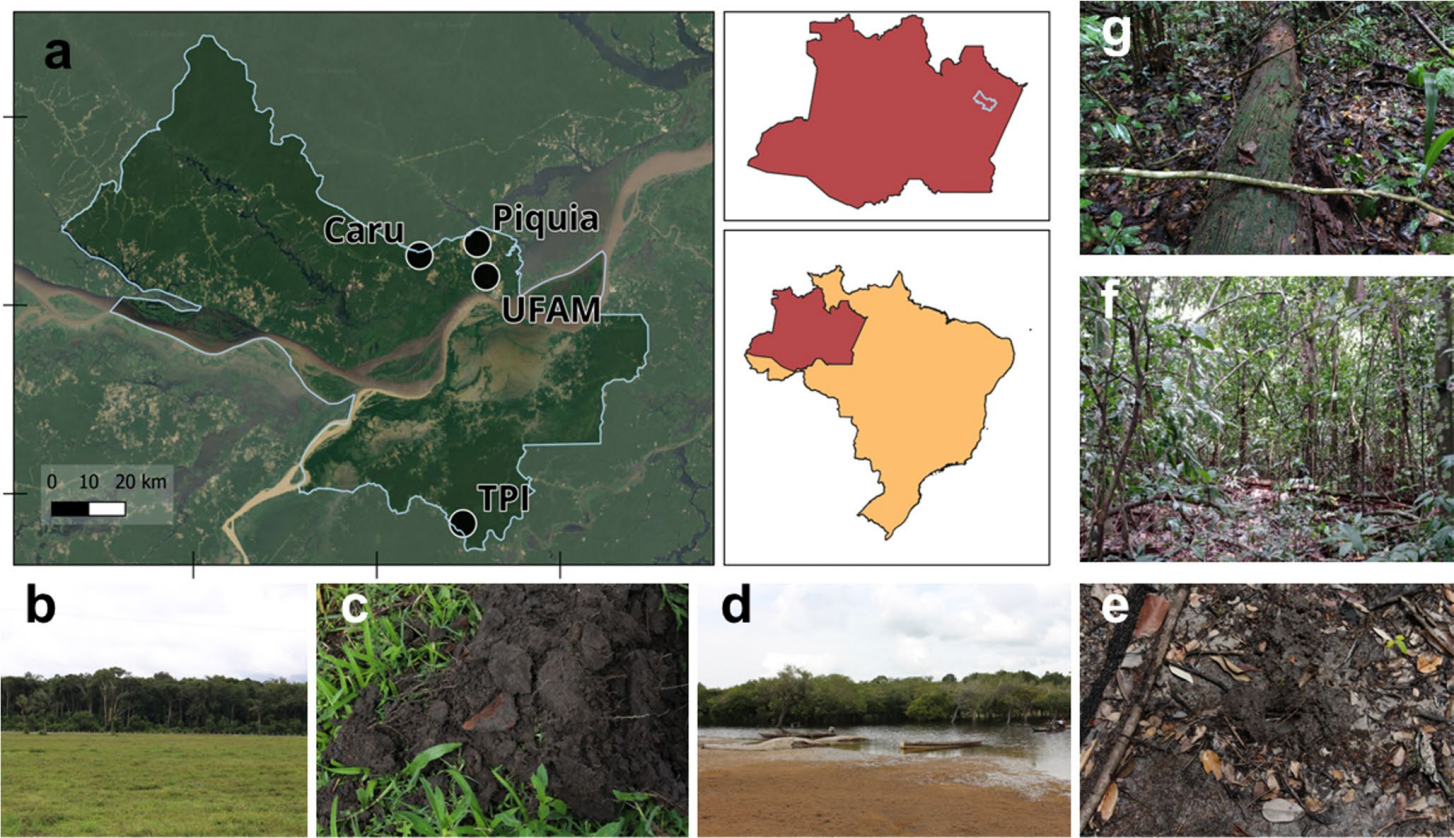
Map of sampling sites (**a**) Location of Itacoatiara municipality, Amazonas state, and the four collection sites. (**b**) *Terra Preta de Indio* (TPI) collection site, a grassy field area with *terra firme* forest at background; (**c**) TPI soil aspect; (**d**) Carú sampling site showing igapó and *Urubu* black water river; (**e**) Carú flooded soil aspect. (**f**) UFAM collection site showing the forest aspect; (**g**) *Piquiá* collection site, showing the forest soil cover. Map produced in Qgis

The study was conducted in an Amazon rainforest biome in the municipality of Itacoatiara, state of Amazonas, Brazil. The sample collections took place in February 2019. Soil samples were collected from four sites within the Amazon Forest biome. Two collection sites were located in two upland forests (high non-flooded ground = *terra firme*), namely “UFAM” (3° 5’ 48.06” S, 58° 27’ 43.35” W) and “Piquiá” (3° 1’ 2.18” S, 58° 29’ 0.64” W). The other two sites were situated in a black-water floodplain forest (*igapó*), namely “Carú” (3° 02’ 47” S, 58° 37’ 31” W), and in an Amazonian dark earth site (*Terra preta de índio*), namely “TPI” (3° 41’ 19.47” S, 58° 31’ 7.62” W) (Fig. 1). At each sample site, the soil surface was scraped to remove plant litter, and approximately 25 g of soil from de top 10 cm were collected every 50 m along a transect, resulting in a total of 20 samples per site and 80 samples in total. Soil samples were placed in sterile plastic bags and kept at 4 °C until processing.

### Yeast isolation and identification

Yeasts were isolated using enrichment and direct isolation methodologies. In the enrichment technique, three subsamples of 1 gram from each soil sample were added to tubes containing 10 ml of Yeast Nitrogen Base (YNB) - glucose medium (YNB 0.67%, glucose 8.0%, chloramphenicol 0.1%). These tubes were incubated separately at 10 °C, 25 °C, and 35 °C for 14 days [10]. When growth was detected, 1 ml of the enriched medium was transferred to a tube containing 5 ml of the corresponding medium and incubated at the same initial temperature for 14 days. Again, when growth was observed, yeast colonies were recovered by plating appropriate decimal dilutions on YM agar (yeast extract 0.3%, malt extract 0.3%, peptone 1%, glucose 2%, chloramphenicol 0.1%, agar 2%), which was also incubated at the same respective temperature of isolation (10 °C, 25 °C, or 35 °C) for 7 days. Representative colonies of the different yeast morphotypes were purified by repeated streaking on YM agar plates and preserved in GYMP broth (glucose 2%, yeast extract 0.5%, malt extract 0.5%, Na2PO4 0.2%) containing 20% glycerol at -80 °C for later identification.

Direct yeast isolation was performed according to Yurkov et al. [9], with a few modifications. One gram of soil was separately suspended in flasks containing 5 ml, 10 ml, or 20 ml of sterile demineralized water and shaken on an orbital shaker for 1 h at 200 rpm. Aliquots of 100 µl from each flask were plated on YM agar (with chloramphenicol 0.02% and ampicillin 0.01%) and incubated for 24 h at 22 °C and then at 4 °C for up to 21 days. Representatives of different colony morphotypes were purified by repeated streak inoculation on YM agar and preserved as described above for later identification.

For the isolation of *Lipomyces* species, one gram of soil was suspended in flasks with 5 ml or 10 ml of sterile demineralized water and shaken on an orbital shaker for 1 h at 200 rpm. Aliquots of 100 µl were plated on Yeast Carbon Base (YCB, Difco, USA) agar supplemented with 0.1% cycloheximide, 0.02% chloramphenicol, and 0.01% ampicillin, and incubated at 25 °C for up to 21 days. Plates were checked after 7, 14, and 21 days of incubation. Colonies were purified by repeated streak inoculation on YCB agar and preserved as described above.

### Yeast identification

For yeast identification, isolates with identical morphology were grouped together and subjected to PCR fingerprinting using the primer (GTG)5 [24]. Yeast isolates with identical PCR fingerprinting patterns were grouped and tentatively considered conspecific [25–27]. At least half of the isolates from each molecular group were identified by sequencing. Species identification was performed by sequencing the ITS-5.8 S region (using primers ITS1 and ITS4) and the D1/D2 variable domains (using primers NL1 and NL4) of the large subunit rRNA gene [28–31]. The amplified DNA was cleaned and sequenced using an ABI 3130 Genetic Analyzer automated sequencing system (Life Technologies, California, USA) with BigDye v3.1 and POP7 polymer. The sequences were edited and aligned using the Muscle program provided in the MEGA7 package [32]. Species identification was performed by comparing the sequences with annotated yeast sequences deposited in the GenBank database using the Basic Local Alignment Search Tool (BLAST at http://www.ncbi.nlm.nih.gov/) [33]. A Neighbor-Joining phylogeny (MEGA6), based on the D1/D2 domains of the large subunit rRNA gene sequences of the *Lipomyces* species, was constructed using 590 aligned positions and the number of substitutions as the distance metric. Bootstrap values were determined from 1,000 replications.

### Enzyme production

The yeast isolates were tested for their ability to degrade starch, protein (casein), pectin, and carboxymethylcellulose, as well as for esterase (from tween 80 hydrolysis) and lipase activities, using the procedures described by Buzzini & Martini [34]. Phosphate solubilization was assessed by the formation of a clear halo on Pikovskaya’s agar medium containing insoluble tricalcium phosphate [35, 36]. Phosphate-solubilizing microorganisms convert insoluble phosphate into a soluble form through the production of organic acids, phosphatases, or other complex compounds. Xylanase activity was tested in YNB agar medium with 1% beechwood xylan as the sole carbon source. Positive xylanase activity was confirmed by the appearance of a clear halo around the colony after staining with iodine solution, which turns the xylan-containing medium a dark gray color. Tannin degradation was tested on plates containing (g/L): tannic acid, 5.0; NaNO_3_, 3.0; KH_2_PO_4_, 1.0; MgSO_4_·7H_2_O, 0.5; KCl, 0.5; FeSO_4_·7H_2_O, 0.01; agar, 30.0; pH 5. A positive test resulted in a clear halo around the colony. For all tests, the inocula consisted of five microliters of a McFarland standard #2 yeast cell suspension, and the plates were incubated at 25 °C for five days.

### Diversity analyses and soil pH measurement

Shannon diversity index and rarefaction analyses were estimated in RStudio v1.2.5042 using the iNEXT package [37]. Venn diagrams were created using jvenn software to analyze the similarity of yeast communities among collection sites, isolation methodologies, and incubation temperatures used for yeast isolation [38]. The pH values of the soil samples were measured according to the protocol described by Teixeira et al. [39].

## Results and discussion

### Yeast diversity in Amazonian rainforest soils

The 80 soil samples collected during this study yielded a total of 598 yeast isolates. Seventy-five ascomycetous and 31 basidiomycetous yeast species, along with four ascomycetous yeast-like fungi species, were found across the four localities, representing a total of 110 species (Table 1). Forty-five candidates for novel yeast species (41% of the identified species) were obtained. Fifty-eight yeast species were isolated as singletons (species found only in one sample), and 18 were doubletons (species found in two samples). Some species isolated as singletons might represent transient species within the soil yeast communities of the sampling sites. The most frequent yeasts obtained from both isolation methods belong to the genera *Apiotrichum*,* Papiliotrema*,* Schwanniomyces*,* Nakaseomyces*,* Saitozyma*, and *Meyerozyma*. Most of the prevalent species were previously reported in studies of forest soil yeasts [3, 9, 14, 40–44]. However, the large number of candidates for novel species highlights the importance of studying culturable yeast soil biodiversity in Amazonian rainforest biomes.

**Table 1:** Yeast species isolated from soil samples collected in four different sites and three temperatures of incubation from the Amazonian rainforest biome

Yeast species	a/b^*^	CARU_10°C	CARU_25°C	CARU_35°C	CARU_D	PIQUIA _10°C	PIQUIA _25°C	PIQUIA _35°C	PIQUIA_D	TPI_10°C	TPI_25°C	TPI_35°C	TPI_D	UFAM_10°C	UFAM_25°C	UFAM_35°C	UFAM_D	Total
*Apiotrichum coprophilum*	*b*	2				3	1		1					8	2		5	22
*Apiotrichum gamsii*	*b*					3								1				4
*Apiotrichum laibachii*	*b*					1			2						1		4	8
*Apiotrichum mycotoxinivorans*	*b*		1													1		2
*Apiotrichum* sp.	*b*				1													1
*Apiotrichum sporotrichoides*	*b*														5			5
*Aureobasidium* sp.	*a*	1		2												2		5
*Candida albicans*	*a*														1	1	1	3
*Candida ghanaensis*	*a*							3								1		4
*Candida leandrae* (*Kodamaea* clade)	*a*									1	1							2
*Candida maltosa* (*Lodderomyces/C. albicans clade*)	*a*							2										2
*Candida natalensis*	*a*								1					1			2	4
*Candida railenensis* (*Kurtzmaniella*)	*a*	3	1			1								1				6
*Candida* sp. 1 (UFMG-CM-Y7123)	*a*						1											1
*Candida* sp. 2 (UFMG-CM-Y7098)	*a*					3	7							1				11
*Candida tropicalis* (*Lodderomyces*/*C. albicans* clade)	*a*											10						10
*Candidozyma* sp. (UFMG-CM-Y7127)	*a*														1			1
*Clavispora* sp. (UFMG-CM-Y7160)	*a*					1												1
*Coniochaeta luteorubra*	*a*			1														1
*Cyberlindnera subsufficiens*	*a*						2								3		2	7
*Diutina* sp. 1 (UFMG-CM-Y7106)	*a*								1									1
*Diutina* sp. 2 (UFMG-CM-Y7108)	*a*								1									1
*Elsinoe* sp.	*a*	1																1
*Filobasidium* sp.	*b*				1												1	2
*Gaillardinia* sp. 1 (UFMG-CM-Y7109)	*a*																1	1
*Galactomyces citri-aurantii*	*a*									1								1
*Geotrichum* sp. 1 (UFMG-CM-Y7113)	*a*						3	4	4			1				1		13
*Geotrichum* sp. 2 (UFMG-CM-Y7119)	*a*						1	2	4		2	2		3				14
*Geotrichum* sp. 3 (UFMG-CM-Y7125)	*a*														1			1
*Groenewaldozyma* sp.	*a*							1										1
*Haglerozyma chiarellii*	*b*														1		2	3
*Hannaella* sp.	*b*									1				1				2
*Hannaella zeae*	*b*									1								1
*Helenozyma melibiosica*	*a*	2							1							1		4
*Kazachstania kunashirensis*	*a*								1									1
*Kazachstania saulgeensis*	*a*					1	4											5
*Kazachstania* sp.	*a*													1				1
*Kazachstania unispora*	*a*									1		2						3
*Kwoniella mangrovensis*	*b*	6													1			7
*Limtongozyma cylindracea*	*a*													1				1
*Limtongozyma* sp.	*a*	1	1															2
*Lipomyces* sp. 1 (UFMG-CM-Y7383)									5									5
*Lipomyces* sp. 2 (UFMG-CM-Y7372)	*a*												1					1
*Lipomyces* sp. 3 (UFMG-CM-Y7369)	*a*												1					1
*Lipomyces* sp. 4 (UFMG-CM-Y7371)	*a*												2					2
*Lipomyces* sp. 5 (UFMG-CM-Y7391)	*a*												5				1	6
*Lipomyces* sp. 6 (UFMG-CM-Y7377)	*a*								1				7					8
*Lipomyces* sp. 7 (UFMG-CM-Y7370)	*a*												2					2
*Lipomyces* sp. 8 (UFMG-CM-Y	*a*								2									
*Lipomyces kononenkoae*	*a*								2									2
*Lipomyces starkeyi*	*a*				4				3								2	9
*Lipomyces yarrowii*	*a*				1				5								3	9
*Martiniozyma asiatica*	*a*					1	1		2									4
*Metahyphopichia laotica*	*a*							1										1
*Metschnikowia koreensis*	*a*					2								1				3
*Metschnikowia* sp. 1 (UFMG-CM-Y7124)	*a*														1			1
*Metschnikowia* sp. 2 (UFMG-CM-Y7163)	*a*																1	1
*Metschnikowia* sp. 3 (UFMG-CM-Y7107)	*a*												2					2
*Meyerozyma caribbica*	*a*	3	3	4	2					2	1	6				1	1	23
*Moesziomyces antarcticus*	*b*												1					1
*Moesziomyces aphidis*	*b*				1													1
*Nakaseomyces nivariensis*	*a*														1			1
*Nakaseomyces* sp.	*a*		4	4				10							4	12		34
*Papiliotrema flavescens*	*b*					1	1							2			1	5
*Papiliotrema laurentii*	*b*	11	1		1	4	1		5	25	13	4	26	1			1	93
*Papiliotrema ruineniae*	*b*					1												1
*Papiliotrema* sp. 1 (UFMG-CM-Y7166)	*b*													2			2	4
*Papiliotrema* sp. 2 (UFMG-CM-Y7118)	*b*										1							1
*Papiliotrema* sp. 3 (UFMG-CM-Y7103)	*b*													1			1	2
*Papiliotrema* sp. 4 (UFMG-CM-Y6631)	*b*										2							2
*Papiliotrema* sp. 5 (UFMG-CM-Y7110)	*b*																1	1
*Papiliotrema terrestris*	*b*								1								3	4
*Pichia manshurica*	*a*							1										1
*Pichia* sp.	*a*					1												1
*Pichia sporocuriosa*	*a*														1			1
*Pichia terrícola*	*a*														1	1		2
*Rhodosporidiobolus ruineniae*	*b*	2								2				1				5
*Rhodotorula mucilaginosa*	*b*	7			1					5	4		2	1		1	1	22
*Saccharomycopsis praedatoria*	*a*						2											2
*Saitozyma flava*	*b*										1							1
*Saitozyma podzolica*	*b*	2	1		10	4			2					6	10		17	52
*Saturnispora diversa*	*a*														1			1
*Scheffersomyces amazonenses*	*a*													2		1		3
*Schwanniomyces polymorphus*	*a*	2	4	2	1	8	6	6	8			1		5	2	1	2	48
*Schwanniomyces* sp.	*a*														1			1
*Schwanniomyces vanrijiae*	*a*							2	2							1	2	7
*Sugiyamaella amazoniana*	*a*							1										1
*Sugiyamaella smithiae*	*a*															2		2
*Sungouiella blattae*	*a*					1												1
*Sungouiella flosculorum*	*a*								1									1
*Sungouiella intermedia*	*a*	1					1											2
*Sungouiella pseudointermedia*	*a*					1		1			3	2						7
*Sungouiella* sp. 1 (UFMG-CM-Y7120)	*a*						1											1
*Sungouiella* sp. 2 (UFMG-CM-Y7165)	*a*								1									1
*Sungouiella* sp. 3 (UFMG-CM-Y7161)	*a*							1										1
*Torulaspora indica*	*a*						1											1
*Torulaspora* sp.	*a*						1											1
*Tremella fuciformis*	*b*				1													1
*Tremella* sp.	*b*																1	1
*Trichosporon asahii*	*b*				1													1
*Trichosporon insectorum*	*b*				1													1
*Vanderwaltozyma polyspora*	*a*				1													1
*Vanrija humicola*	*b*	1	1		2	1					1						2	8
*Wickerhamiella* sp.	*a*						1											1
*Wickerhamomyces lynferdii*	*a*	1	1															2
*Wickerhamomyces* sp.	*a*					1												1
*Wickerhamomyces sydowiorum*	*a*		2															2
*Xenoacremonium falcatus*	*a*			1														1
*Yamadazyma* sp. 1 (UFMG-CM-Y6633)	*a*					5	4		3									12
*Yamadazyma* sp. 2 (UFMG-CM-Y7115)	*a*							1										1
*Yamadazyma* sp. 3 (UFMG-CM-Y7117)	*a*							1										1

^*^ a = Ascomycetous yeast, b = Basidiomycetous yeasts

Using the direct isolation methodology, 150 yeast isolates were obtained, representing 40 species (Table 1). *Papiliotrema laurentii*,* Sa. podzolica*, and *Schwanniomyces polymorphus* were the most prevalent species, representing 22%, 19%, and 7% relative abundance, respectively. Other yeast species did not reach 5% relative abundance. The UFAM site had the highest number of isolates (54), followed by “Piquiá” (41), TPI (31), and “Carú” (24). UFAM was also the site from which the greatest number of species was isolated (22 species), followed by “Piquiá” (18), “Carú” (13), and TPI (4). Only *Pa*. *laurentii* was recovered from all collection sites using the direct isolation technique. The average values of the total yeast counts, in CFU/g per gram of soil, were 1.1 × 10³ for “Carú,” 2.1 × 10³ for the “TPI”, 1.9 × 10⁴ for “Piquiá,” and 4.2 × 10⁴ for UFAM.

A total of 401 yeast isolates were obtained using the enrichment technique. These isolates were identified as belonging to 81 species, with 21 and 60 species having basidiomycetous and ascomycetous affinities, respectively. Four yeast-like fungi were also isolated (Table 1). A high prevalence of singletons was observed, with 36 species represented by a single isolate, corresponding to 44% of the yeasts obtained with this methodology. Ascomycetous yeasts were prevalent in all collection sites, and the most frequent species were *Sch. polymorphus*, with 37 isolates and a relative abundance of 9.2%, and *Nakaseomyces* sp., with 34 isolates and a relative abundance of 8.5%. Among basidiomycetous species, *Pa. laurentii* (60 isolates, 15% relative abundance) and *Sa. podzolica* (23 isolates, 5.7% relative abundance) were the most frequent. Other ascomycetous and basidiomycetous species had relative abundances lower than 5%. Eight yeast-like fungi were recovered, belonging to the genera *Aureobasidium* (five isolates), *Coniochaeta*,* Elsinoe*, and *Xenoacremonium* (one isolate each). Together, all yeast-like fungi species had only 2% abundance in the community. Twenty-eight candidates for possible new species were obtained using this methodology. With the enrichment technique, the number of ascomycetous species was higher than that of basidiomycetous species. In contrast, with the direct isolation procedure, the number of species from these two phyla was identical (20 species each); however, the number of basidiomycetous yeast isolates (102 out of 150 isolates) corresponded to 68% of the total isolates using the direct isolation methodology.

Enrichment techniques and sugar-rich media can favor the isolation of fast-growing or fermentative ascomycetous yeasts [45, 46]. Nevertheless, in our work, almost all basidiomycetous species isolated by the direct isolation technique were also isolated by the enrichment procedure. Exceptions were *Filobasidium* sp., *Moesziomyces antarcticus*,* Papiliotrema terrestris*, and six other singleton species that were isolated only by the direct isolation technique. Basidiomycetous species are generally dominant in forest soils in boreal and temperate regions [8, 44]. Previous studies on soil yeast diversity conducted in Brazil showed that ascomycetous species tend to be prevalent in soil from tropical regions. Vital et al. [12] found a prevalence of 82% of ascomycetous species in soil samples collected from sites in the Amazonian rainforest in the state of Roraima. Soil samples collected in Cerrado biomes in Southeastern Brazil had an 80% prevalence of Ascomycota species when soil suspensions were plated on solid medium [13]. Ascomycetous species corresponded to 79% of all 37 yeast species isolated by Moreira and Vale [15] from soil samples collected in Cerrado and Atlantic Forest biomes in Brazil. In this case, the authors used liquid sugar-rich media as a substrate for yeast isolation. A culture-independent approach (ITS2 metabarcoding) revealed a prevalence of 78.7% Ascomycota in total fungal sequences (yeasts and filamentous fungi) in soil samples collected in the Atlantic Forest, iron outcrops, eucalyptus plantations, and revegetated areas with grass in an iron-mining site in central Brazil [47]. However, considering only yeast sequences, 59.5% were assigned to Basidiomycota and 36.5% to Ascomycota [47]. Dunthorn et al. [48] found a prevalence of 63.8% Saccharomycotina sequence reads in soil samples collected from three Neotropical rainforests located in Costa Rica (La Selva Biological Station), Panama (Barro Colorado Island), and Ecuador (Tiputini Biodiversity Station). The authors suggest that due to frequent precipitation, the constant anoxic conditions found in tropical soils could favour yeast dominance in the soil fungal community. Therefore, soil yeast abundance and species composition can differ depending on vegetation type and land management [44, 49]. The high plant species richness and diversity in the Amazonian rainforest biomes studied, differences in soil chemistry at each collection site, and the different yeast isolation methodologies may explain the predominance of ascomycetous species in our work. The pH values of the soil samples from these collection sites were similar, ranging from 5.3 for “Piquiá,” 5.2 to 5.4 for UFAM, 5.6 to 5.9 for TPI, and 6.1 to 6.2 for “Carú.” These soils could be considered acidic, and the pH values could be considered appropriate for yeast growth.

### The use of selective media for *Lipomyces* species isolation

**Fig. 2:**
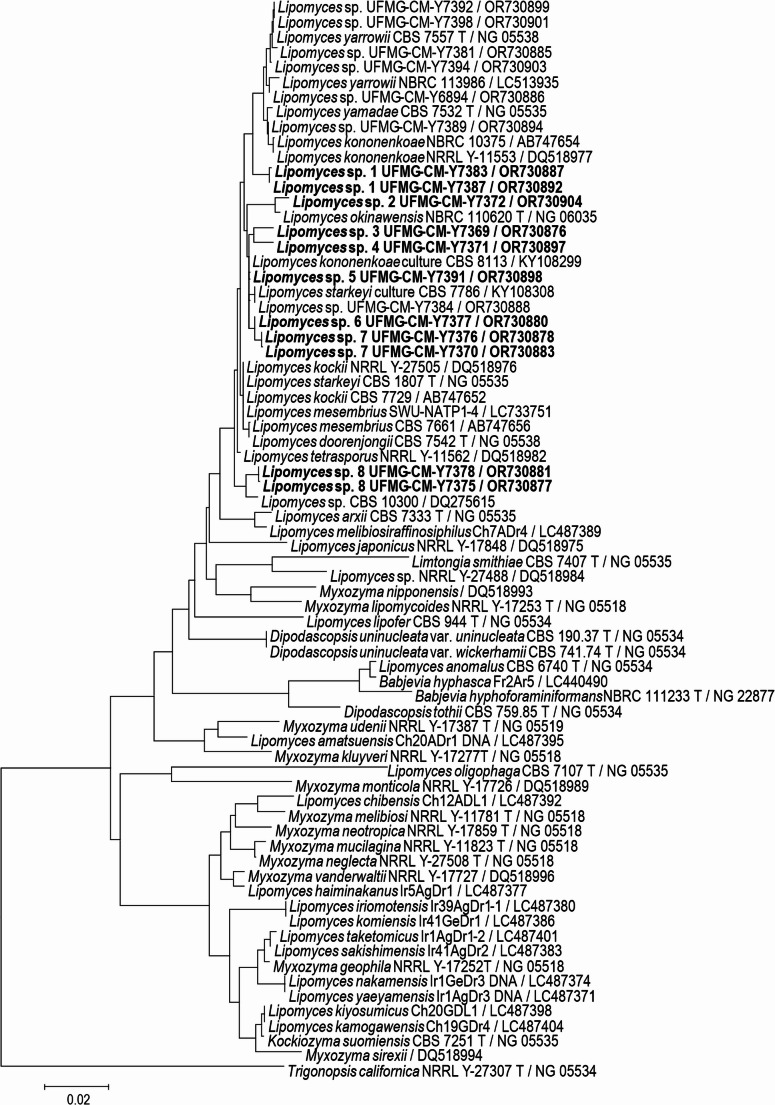
Neighbor-Joining phylogram of *Lipomyces* and related species based on a 590-position alignment of the D1/D2 domains of the LSU rRNA gene, showing the placement of possible new species (marked with different colours) recovered in this study. The distance metric is the number of differences. Gaps were deleted on a pairwise basis. Bootstraps determined from 1000 pseudoreplications are shown for value of 50% and greater

Forty-seven *Lipomyces* isolates were obtained from 36 out of 80 soil samples collected. The isolates were only obtained when YCB medium with cycloheximide and antibiotics was used. Total counts of *Lipomyces* spp. in soil samples ranged from 5.0 × 10¹ to 1.1 × 10³ CFU g⁻¹ for “TPI”; 5.0 × 10¹ to 2.4 × 10³ CFU g⁻¹ for “UFAM”; 5.0 × 10¹ to 7.5 × 10² CFU g⁻¹ for “Carú”; and 5.0 × 10¹ to 2.0 × 10² CFU g⁻¹ for “Piquiá.” The number of *Lipomyces* isolates was 19, 17, six, and five for “Piquiá,” TPI, “Carú,” and UFAM, respectively. The prevalent *Lipomyces* species were *L. starkeyi* and *L*. *yarrowii*. Based on D1/D2 sequences, most of the *Lipomyces* isolates were grouped into eight candidates for new species (Fig. 2). These Amazonian *Lipomyces* species were phylogenetically related to species of a subclade containing *Lipomyces kononenkoae*,* L. okinawensis*,* L. starkeyi*,* L. yamadae*, and *L. yarrowii*. However, further studies using other sequences, such as EF-1α, or whole-genome sequencing are necessary to confirm the species identity of these isolates. *Lipomyces* and phylogenetically related genera (*Dipodascopsis* and *Myxozyma*) are oleaginous species known as true soil inhabitants with a possible worldwide distribution [50]. These yeasts were not isolated using the enrichment and direct isolation techniques in our work, highlighting the necessity of using selective media for the isolation of these species [50–53].

### Richness and diversity index values for the soil yeast communities

**Fig. 3:**
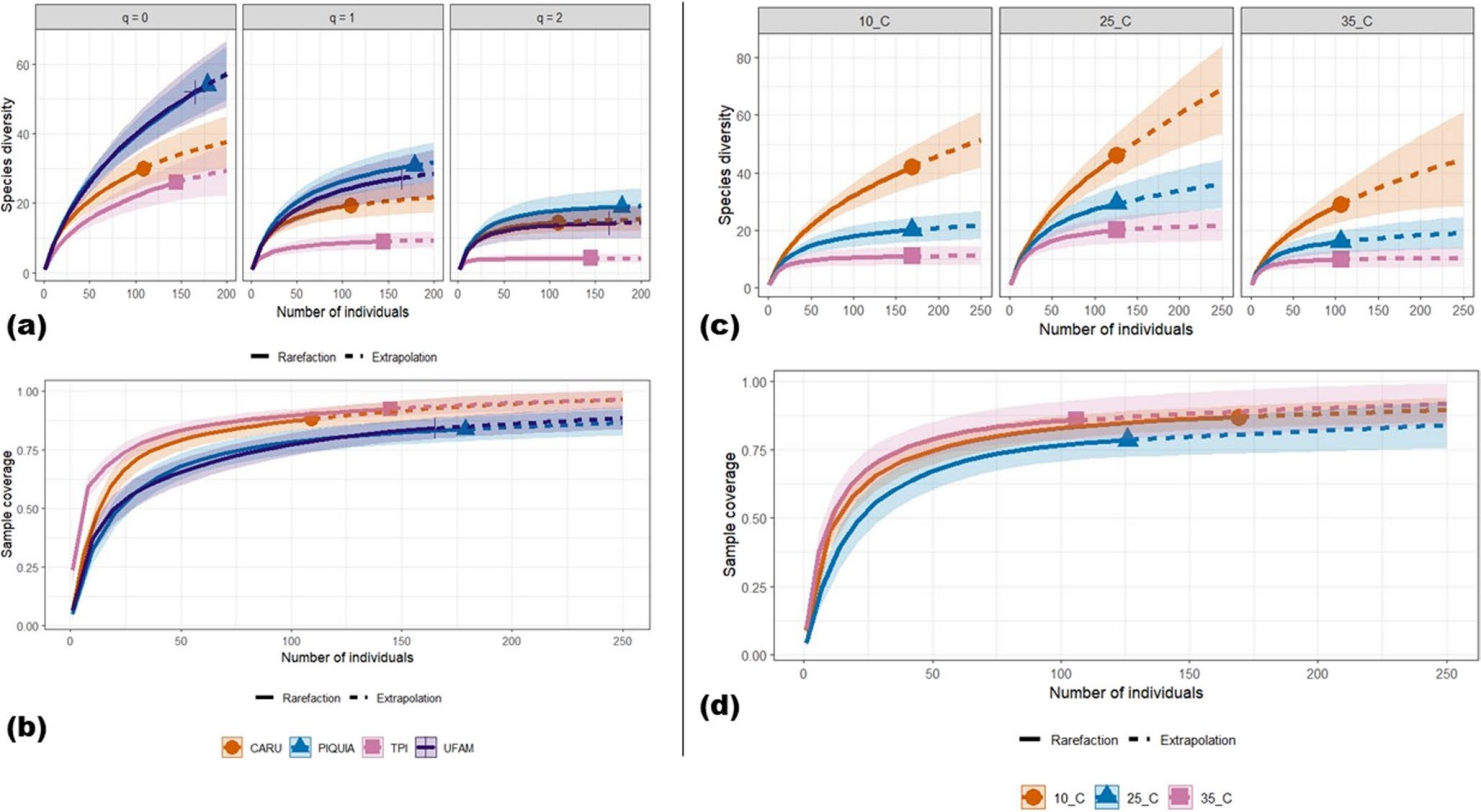
Diversity of yeasts isolated from soil samples by enrichment isolation in liquid media. **a** Sample-size-based rarefaction (solid line segment) and extrapolation (dotted line segments) sampling curves with 95% confidence intervals (shaded areas) for yeast communities isolated from soil samples from four collection sites (Carú, TPI, Piquiá and UFAM) in Itacoatiara-AM, separately by diversity order: q = 0 (species richness), q = 1 (Shannon diversity) and q = 2 (Simpson diversity). The solid dots/triangles/ circles/pipe represent the collection sites. **b** Sample completeness curves for the yeast communities from four sampling sites. **c** Sample-size-based rarefaction and extrapolation for yeast communities isolated from soil samples incubated in three different isolation temperatures. The solid dots/triangles/ circles/pipe represent different isolation temperatures. **d** Sample completeness curves for the yeast communities obtained from three different incubation temperatures

**Fig. 4:**
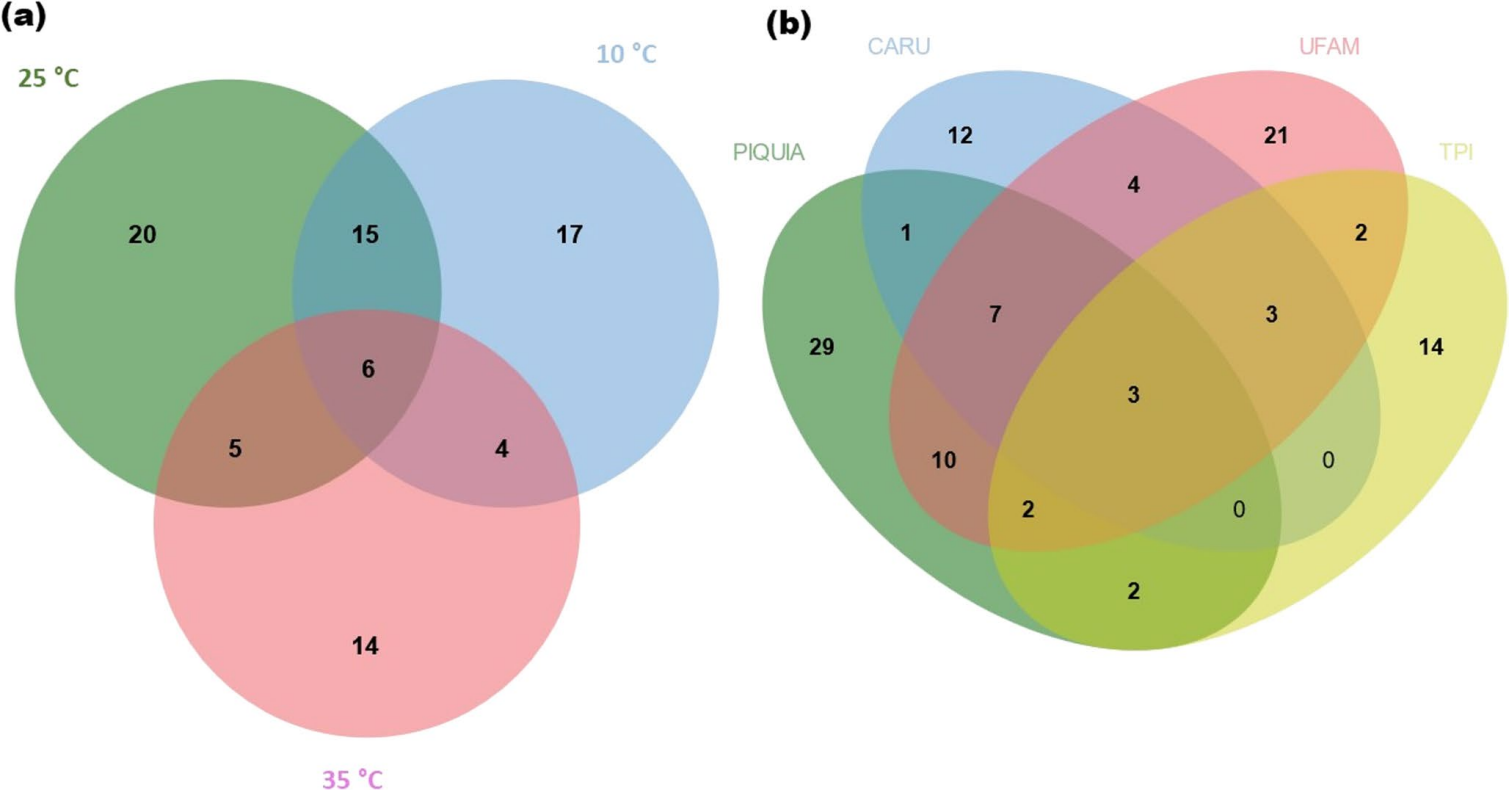
- Venn diagrams showing the number of exclusive and shared species for different isolation temperature in enrichment isolation technique (**a**) and different collection sites (**b**)

“UFAM” and “Piquiá” had the highest species richness and Shannon diversity index values, followed by “Carú” and “TPI.” The collection effort was sufficient to describe 92% of the estimated species richness in “TPI,” 88% in “Carú,” and 84% in “Piquiá” and “UFAM.” Sample-size-based extrapolation of yeast species diversity based on Hill numbers estimates that a collection effort larger than 218 isolates for “Carú” and “TPI” sites and larger than 358 for “Piquiá” and “UFAM” is needed to achieve 90% sample coverage. A comparison of yeast species present in soil samples collected from all four sampling sites revealed low similarity in community species composition, with a great number of singletons and only three species (*Pa. laurentii*,* Sch. polymorphus*, and *Va. humicola*) shared among the collection sites (Fig. 3 and 4). According to Yurkov [3] and Yurkov et al. [9, 49], yeast diversity between sample sites is irregularly distributed, with low species richness at a single site, few species shared between sampled sites, and a greater number of yeast species when considering a forest or a region. As expected, yeast community composition was more similar between “Piquiá” and “UFAM” sites, which present similar forest coverage (upland “terra-firme” forest) (Fig. 3). These sites also had similar species richness, Shannon, and Simpson diversity indexes (Table S1). The yeast community in the “TPI” site had the lowest species richness, Shannon, and Simpson diversity index values (Fig. 3; Table S1). This collection site was located in a grassy field area, without forest-type vegetation cover. Considering that vegetation and other above-ground substrates influence soil yeast diversity, low vegetation cover could explain the low diversity of soil yeast communities at this site. The “Carú” collection site was located on an igapó forest ecotone. This type of forest consists of a diverse set of specific plant species growing along black water rivers with high humic acid concentrations [20]. This unique vegetation may be responsible for the species richness in “Carú,” as well as the differences in Shannon and Simpson diversity indexes compared to the “terra firme” yeast communities. Additionally, all yeast species found in “Carú” were obtained from soil samples collected in waterlogged areas. It is possible that these yeast species could survive and grow under micro-aerobic conditions, as observed by Jaiboon et al. [54] for yeasts from peat in a tropical peat swamp forest in Thailand.

### Production of extracellular enzymes by the yeast isolates

Three hundred and fourteen yeast isolates were evaluated, and 231 showed at least one extracellular enzymatic activity (Table 2). Twenty-three isolates, belonging to 16 species, did not show any enzymatic activity. Xylanase was the most prevalent extracellular enzymatic activity observed among the yeast isolates tested (positive in 50.6% of the isolates), followed by pectinase (41.1%), lipase (37.3%), esterase (32.5%), protease (29.6%), amylase (22.9%), tannase (18.5%), and cellulase (11.8%). *Papiliotrema laurentii* was the only species able to produce all the tested enzymes. The ability to solubilize phosphate was observed in 45% of the isolates tested. These results suggest that the soil yeast isolates can contribute to the decomposition and mineralization of organic matter and nutrients in this Amazonian rainforest biome. Enzymatic activities were evaluated for soil yeasts collected in the Cerrado biome by Carvalho et al. [13]. These authors detected xylanase and cellulase activities in 7.8% and 5.8% of total isolates tested, respectively, while esterase was detected in all tested strains. In our study, xylanase and cellulase were expressed by 50.5% and 11.9%, respectively, of all isolates tested, suggesting that these yeasts contribute to the decomposition of lignocellulosic materials in these soils (Fig. 4).

**Table 2:** – Enzymatic activities of the yeast isolates obtained from the Amazonian rainforest biome

Yeast species	Number of isolates tested for enzimatic activity	AmA	LipA	EstA	ProA	XylA	CelA	PecA	PhoA	TanA
*Apiotrichum coprophilum*	9	9	1	0	3	9	9	0	0	7
*Apiotrichum gamsii*	2	2	0	0	0	2	2	0	0	1
*Apiotrichum laibachii*	5	2	1	0	4	5	5	1	0	2
*Apiotrichum mycotoxinivorans*	2	0	1	1	0	1	1	0	0	0
*Apiotrichum sp.*	1	1	0	1	0	1	1	0	0	0
*Apiotrichum sporotrichoides*	3	3	1	0	0	3	3	2	1	2
*Aureobasidium*	4	3	1	1	1	3	1	2	0	0
*Candida albicans*	3	0	2	3	0	0	0	1	3	0
*Candida ghanaensis*	3	0	0	0	0	0	0	0	0	0
*Candida leandrae*	2	0	0	1	0	0	0	0	1	0
*Candida maltosa*	2	0	2	2	0	0	0	0	0	0
*Candida natalensis*	3	0	1	0	0	0	0	0	0	1
*Candida nivariensis*	1	0	0	0	0	0	0	0	1	0
*Candida railenensis*	3	0	1	0	0	1	0	0	3	0
*Candida* sp. 1	1	0	0	0	0	0	0	0	1	0
*Candida* sp. 2	7	0	0	1	1	1	0	0	6	1
*Candida tropicalis*	4	0	2	4	0	3	0	0	3	0
*Candidozyma* sp.	1	0	0	1	0	0	0	0	0	0
*Clavispora* sp.	1	0	0	0	0	0	0	0	0	0
*Coniochaeta luteorubra*	1	1	1	1	0	1	0	0	0	0
*Cyberlindnera subsufficiens*	3	0	0	0	1	0	0	1	0	0
*Diutina* sp. 1	1	0	0	0	0	0	0	1	0	0
*Diutina* sp. 2	1	0	1	0	0	0	0	1	0	0
*Elsinoe* sp.	1	0	0	1	0	1	0	1	1	1
*Filobasidium* sp.	2	0	1	2	2	0	1	1	1	0
*Gaillardinia* sp.	1	0	0	1	0	1	0	0	0	0
*Galactomyces citri-aurantii*	1	0	0	0	0	0	1	0	0	0
*Geotrichum* sp. 1	7	0	0	0	0	0	1	0	0	1
*Geotrichum* sp. 2	7	0	0	0	0	1	3	0	0	0
*Geotrichum* sp. 3	1	0	0	0	0	0	0	0	0	0
*Groenewaldozyma* sp.	1	0	0	0	0	0	0	0	1	0
*Haglerozyma chiarellii*	2	0	1	2	0	2	0	2	0	0
*Hannaella* sp.	2	0	1	0	0	1	0	1	2	0
*Hannaella zeae*	1	0	0	0	0	1	0	1	1	0
*Helenozyma melibiosica*	3	0	1	0	0	0	0	0	1	0
*Kazachstania kunashirensis*	1	0	1	0	0	0	0	1	1	0
*Kazachstania saulgeensis*	3	0	0	0	0	1	0	0	3	0
*Kazachstania* sp.	1	0	0	0	0	0	0	0	1	0
*Kazachstania unispora*	2	0	0	0	0	0	0	0	2	0
*Kwoniella mangrovensis*	4	1	0	0	1	4	1	3	2	1
*Limtongozyma cylindracea*	1	0	0	1	0	0	0	0	1	0
*Limtongozyma sp.*	1	0	0	0	0	0	0	1	0	0
*Lipomyces* sp. 1 (UFMG-CM-Y7383)	4	4	4	0	4	4	0	4	4	0
*Lipomyces* sp. 2 (UFMG-CM-Y7372)	1	1	1	0	1	1	0	1	1	0
*Lipomyces* sp. 3 (UFMG-CM-Y7369)	1	1	1	0	1	1	0	1	1	0
*Lipomyces* sp. 4 (UFMG-CM-Y7371)	2	2	2	0	2	2	0	0	2	0
*Lipomyces* sp. 5 (UFMG-CM-Y7391)	5	0	5	0	4	4	0	4	5	0
*Lipomyces* sp. 6 (UFMG-CM-Y7377)	3	3	3	0	3	3	0	3	3	0
*Lipomyces* sp. 7 (UFMG-CM-Y7370)	2	2	2	0	2	2	0	2	2	0
*Lipomyces kononenkoae*	1	1	1	0	1	1	0	1	1	0
*Lipomyces starkeyi*	8	8	8	0	8	8	0	5	8	0
*Lipomyces yarrowii*	9	8	9	0	9	9	0	6	9	0
*Martiniozyma asiatica*	2	0	1	0	0	0	0	0	2	0
*Metahyphopichia laotica*	1	0	0	0	0	0	0	0	1	1
*Metschnikowia koreensis*	2	0	0	2	2	0	0	1	2	0
*Metschnikowia* sp. 1	1	0	0	0	0	0	0	0	0	0
*Metschnikowia* sp. 2	1	0	0	0	1	0	0	1	1	0
*Metschnikowia* sp. 3	2	0	0	1	0	1	0	0	2	1
*Meyerozyma caribbica*	10	0	3	0	0	0	0	0	9	1
*Moesziomyces antarcticus*	1	1	0	1	0	1	0	1	0	1
*Moesziomyces aphidis*	1	1	1	0	0	1	1	1	0	0
*Nakaseomyces* sp.	14	0	0	1	0	1	0	0	14	0
*Papiliotrema flavescens*	5	1	4	5	5	3	0	4	4	1
*Papiliotrema laurentii*	34	11	13	30	14	33	3	30	6	21
*Papiliotrema ruineniae*	1	0	1	1	1	1	0	1	1	0
*Papiliotrema* sp. 1	3	0	2	3	1	1	0	1	1	3
*Papiliotrema* sp. 2	1	0	0	0	1	1	0	0	1	0
*Papiliotrema* sp. 3	2	0	0	2	2	2	2	2	0	1
*Papiliotrema* sp. 4	2	0	2	2	2	1	0	1	1	0
*Papiliotrema* sp. 5	1	0	0	1	1	1	0	1	1	1
*Papiliotrema terrestris*	2	0	2	2	1	0	0	2	2	0
*Pichia* sp.	1	0	1	0	0	0	0	0	1	0
*Pichia manshurica*	1	0	1	0	0	0	0	0	0	0
*Pichia sporocuriosa*	1	0	0	0	0	0	0	0	1	0
*Pichia terricola*	1	0	0	0	0	0	0	0	1	0
*Rhodosporidiobolus ruineniae*	3	0	1	3	0	1	0	3	0	0
*Rhodotorula mucilaginosa*	9	1	0	0	3	1	0	8	1	0
*Saccharomycopsis praedatoria*	2	0	0	0	1	1	0	2	2	0
*Saitozyma flava*	1	1	0	1	0	1	0	1	1	0
*Saitozyma podzolica*	13	1	6	12	0	13	0	11	0	4
*Saturnispora diversa*	1	0	0	0	0	0	0	0	1	0
*Scheffersomyces amazonensis*	3	0	1	0	0	0	0	0	2	0
*Schwanniomyces polymorphus*	12	1	5	0	0	11	0	0	0	0
*Schwanniomyces* sp.	1	0	1	0	1	1	0	0	1	0
*Schwanniomyces vanrijiae*	4	0	3	0	0	0	0	3	0	0
*Sugiyamaella smithiae*	2	0	2	0	0	0	0	0	0	0
*Sugiyamaella* sp.	1	0	0	0	0	0	0	0	0	0
*Sungouiella flosculorum*	1	0	1	0	0	0	0	0	1	0
*Sungouiella pseudointermedia*	5	0	0	0	0	0	0	0	2	0
*Sungouiella* sp. 1	1	0	0	0	0	0	0	0	0	0
*Sungouiella* sp. 2	1	0	1	1	0	0	0	0	1	0
*Sungouiella* sp. 3	1	0	1	0	0	0	0	0	1	0
*Torulaspora indica*	1	0	0	0	0	0	0	1	1	0
*Torulaspora* sp.	1	0	0	1	1	0	0	0	1	0
*Tremella fuciformis*	1	0	0	0	0	0	0	1	0	1
*Tremella* sp.	1	0	0	1	0	0	0	1	0	0
*Trichosporon asahii*	1	0	1	1	0	0	1	1	0	1
*Trichosporon insectorum*	1	0	1	0	0	0	0	0	0	0
*Vanderwaltozyma polyspora*	1	0	1	0	0	0	0	1	0	1
*Vanrija humicola*	4	0	3	4	3	1	1	1	0	0
*Wickerhamiella* sp.	1	0	0	1	0	0	0	0	1	0
*Wickerhamomyces* sp.	1	0	0	0	1	0	0	0	1	0
*Wickerhamomyces sydowiorum*	2	1	0	1	2	2	0	0	2	2
*Xenoacremonium falcatus*	1	1	0	1	0	1	0	1	0	1
*Yamadazyma* sp. 1	4	0	1	0	2	1	0	1	0	0
*Yamadazyma* sp. 2	1	0	0	0	0	0	0	0	0	0
*Yamadazyma* sp. 3	1	0	1	1	0	1	0	0	1	0
Total strains	314									
Total of positive strains		72	117	102	93	159	37	129	143	58
Percentage of positive on total strains (%)		22.9	37.3	32.5	29.6	50.6	11.8	41.1	45.5	18.5

*AmA* Amylase activity, *LipA* Lipase activity, *EstA* Esterase activity (hydrolisys of tween 80), *XylA* Xylanase activity, *CelA* Cellulase activity (degradation of carboxymethyl-cellulose), *PecA* Pectinase activity, *PrA* Protease activity, *TanA* Tannase activity

Several yeast species, including *Saccharomyces*, *Candida*, *Saccharomycopsis*, *Aureobasidium*, *Rhodotorula*, *Kluyveromyces*, *Geotrichum*, *Wickerhamomyces*, and former *Cryptococcus* species, have been reported as capable of degrading pectin [55, 56]. In our study, isolates of *Apiotrichum*, *Haglerozyma*, *Kwoniella*, *Lipomyces*, *Rhodosporidiobolus*, and *Schwanniomyces* also exhibited pectinase production. Lipases were notably expressed by isolates of *Lipomyces*, *Papiliotrema*, and *Schwanniomyces*, and species of these genera have been reported as lipase-producers [54, 57]. The high number of lipase-producers obtained in our work suggests these yeasts perform an important role in the digestion of lipids in these Amazonian soils, contributing for the nutrient cycling in these environments. Esterase activity, from tween 80 hydrolysis, was observed in 39 out of 107 tested species, with *Pa. laurentii* and *Sa. podzolica* exhibiting the highest number of isolates positive for this enzyme. Carvalho et al. [13] also explored esterase activity in soil yeasts isolated from the Cerrado ecosystem in Brazil. All yeast isolates tested by Carvalho et al. [13] were able to produce esterase activity, and the highest enzymatic activities were observed for *Candida* sp., *C. neerlandica*, *C. tetrigidarum*, *C. humicola*, *K. exigua*, *P. kudriavzevii*, *S. pseudopolymorphus*, *S. vanrijiae*, and *T. loubieri*. These authors also tested the soil yeast strains for cellulase production using CMC as a substrate. Cellulase production was observed in 18 out of 307 isolates tested. These isolates were identified as *Cryptococcus laurentii*, *Cr. flavescens*, *Cr. humicola*, and *C. neerlandica*. In our work, cellulase was the least expressed enzyme, only occurring in *Aureobasidium*, *Filobasidium* sp., *G. citri-aurantii*, two candidates for new *Geotrichum* species, *K. mangrovensis*, *M. aphidis*, *Papiliotrema* sp. 3, *P. laurentii*, *Tr. asahii*, *V. humicola*, and six *Apiotrichum* species. Notably, only three of 34 tested strains of *P. laurentii* produced cellulase. We also tested the isolates’ capacity to produce amylase from starch. Amylase production was widespread in *Lipomyces* and *Apiotrichum* species, particularly in *A. coprophilum*, with all nine isolates tested exhibiting amylase activity. Previous works reported tannase production only by *Candida* sp., *C. parapsilosis*, *C. tropicalis*, *Candida utilis* (= *Cyberlindnera jadinii*), *Aureobasidium* spp., *Blastobotrys adeninivorans*, *Kluyveromyces marxianus*, *Rhodotorula diobovatum*, *Debaryomyces hansenii*, *P. kudriavzevii*, *P. kluyveri*, *P. terricola*, *Komagataella pastoris*, and *Sporidiobolus ruineniae* [58–64]. In our study, tannase production was observed in 24 different yeast species, notably within the genera *Apiotrichum* and *Papiliotrema*, as well as 13 other genera. These enzymes contribute to the degradation of plant tannins present in soil. Hydrolysis of tannic acid by tannase results in the liberation of glucose, gallic acid and various galloyl esters of glucose [63], contributing for soil fertility. In this work, *P. laurentii* was the only species able to produce all tested enzymes. The enzymatic activity profile displayed by *Pa. laurentii* likely gives this species an ecological advantage in soil colonization, as it was also the only species isolated from all collection sites by both direct and enrichment isolation methodologies. Additionally, the extracellular enzymatic profile of Amazonian soil yeasts suggests that these microorganisms contribute to the recycling of organic matter in this forest biome.

Microorganisms that can solubilize phosphorus play a role in plant growth promotion and productivity by enhancing the availability of this mineral in soil [65, 66]. Phosphorus solubilization by microorganisms occurs through the release of substances such as organic acids and enzymes like phosphatases [66]. Species of *Candida*, *Geotrichum*, *Rhodotorula*, and *Cyberlindnera* are known to solubilize inorganic phosphate [1]. In our work, we observed the ability to solubilize phosphate in several isolates of *Hannaella*, *Kazachstania*, *Kwoniella*, *Lipomyces*, *Metschnikowia*, *Meyerozyma*, *Nakaseomyces*, *Papiliotrema*, and *Pichia*. These isolates may contribute to the solubilization of inorganic phosphorus for plant growth in this forest biome.

### Candidates for novel yeast species

Forty-five potential candidates for new yeast species were found in our study, most of which were represented by singletons (Table 3). Novel yeast species have also been observed in other studies in soils of temperate and Mediterranean regions [9, 40, 49, 67], demonstrating that soil can be a tremendous reservoir for the discovery of new yeasts. Here, we will discuss only the occurrence of the novel species that were reported at relatively high frequencies. *Nakaseomyces* sp., represented by 34 isolates, was recovered from the “Carú,” “Piquiá,” and UFAM sites, and only through the enrichment procedure. D1/D2 sequences identical or very similar (1–3 nucleotide differences) to this species are deposited in the GenBank database. These sequence deposits belong to yeasts collected from tree bark and rotting wood in Brazil, forest soil collected in Thailand, and leaves from Taiwan. This species was also isolated from soil samples collected in native and reforested areas at a post-mining site, along Cerrado and Atlantic Forest biomes in southeastern Brazil [15]. Based on this, we can infer that this species has a widespread distribution and is primarily associated with plants, mainly in tropical biomes, being then transferred to the soil by plant detritus.

**Table 3:** Candidates for novel yeast species isolated from soil samples collected in Amazonian Rainforest biomes based on D1/D2 and ITS sequences

Species	UFMG code	Region	Genbank access	Closest described species	Strain	Genbank access	Identity	Gaps
*Apiotrichum* sp.	UFMG-CM-Y7112	**D1/D2**	OM321343	*Apiotrichum dehoogii*	CBS 8686	NG_066149	511/542(94%)	3/542(0%)
*Candida* sp. 1	UFMG-CM-Y7123	**D1/D2**	OM321356	*Candida californica*	CBS:989	KY106378	499/525(95%)	0/525(0%)
*Candida* sp. 2	UFMG-CM-Y7098	**D1/D2**	OM321328	*Candida thaimueangensis*	NRRL Y-27,416	EF550231	454/529(86%)	20/529(3%)
*Candidozyma* sp.	UFMG-CM-Y7127	**D1/D2**	OM321360	*Candidozyma ruelliae*	MTCC 7739	NG_064309	416/491(85%)	22/491(4%)
		**ITS**	OR228682	*Sungouiella ezoensis*	CBS:11,753	KY102082	337/367(92%)	0/525(0%)
*Clavispora* sp.	UFMG-CM-Y7160	**D1/D2**	OR211598	*Clavispora fructus*	CBS:6380	KY106454	502/527(95%)	3/527(0%)
		**ITS**	OR228683	*Clavispora asparagi*	CBS 9770	NR_155004	343/373(92%)	16/373(4%)
*Diutina* sp. 1	UFMG-CM-Y7106	**D1/D2**	OM321336	*Diutina sipiczkii*	NCAIM Y.02231	MN219653	400/461(87%)	13/461(2%)
*Diutina* sp. 2	UFMG-CM-Y7108	**D1/D2**	OM321338	*Diutina siamensis*	DMKU-RE43	KT336715	408/427(96%)	2/427(0%)
*Elsinoe* sp.	UFMG-CM-Y7104	**D1/D2**	OM321334	*Elsinoe banksiigena*	CPC 32,402	NG_064552	500/533(94%)	2/533(0%)
		**ITS**	OM480728	*Myriangium duriaei*	CBS:260.36	MH855793	434/514(84%)	30/514(5%)
*Filobasidium* sp.	UFMG-CM-Y6635	**D1/D2**	OM321340	*Filobasidium mali*	CGMCC2.4012	MK050346	574/574(100%)	0/574(0%)
		**ITS**	OM480729	*Filobasidium globosum*	CGMCC2.5680	MK050344	593/607(98%)	2/607(0%)
				*Filobasidium mali*	CGMCC2.4012	MK050346	581/610(95%)	11/610(1%)
*Gaillardinia* sp.	UFMG-CM-Y7109	**D1/D2**	OM321339	*Gaillardinia entomophila*	NRRL Y-7783	NG_055237	433/512(85%)	38/512(7%)
*Geotrichum* sp. 1	UFMG-CM-Y7113	**D1/D2**	OM321344	*Galactomyces geotrichum*	CBS 775.71	JN974280	482/494(98%)	1/494(0%)
*Geotrichum* sp. 2	UFMG-CM-Y7119	**D1/D2**	OM321351	*Geotrichum europaeum*	CBS 866.68	NG_055387	480/494(97%)	0/494(0%)
*Geotrichum* sp. 3	UFMG-CM-Y7125	**D1/D2**	OM321358	*Galactomyces pseudocandidus*	UCDFST 01-150	MH130227	470/480(98%)	0/480(0%)
*Groenewaldozyma* sp.	UFMG-CM-Y7164	**D1/D2**	OR211600	*Groenewaldozyma tartarivorans*	CBS 7955	NG_058300	525/556(94%)	5/556(0%)
		**ITS**	OR228685	*Groenewaldozyma auringiensis*	CBS:6920	KY103490	374/398(94%)	6/398(1%)
*Kazachstania* sp.	UFMG-CM-Y7101	**D1/D2**	OM321331	*Kazachstania yasuniensis*	CLQCA 20–132	NG_067774	573/574(99%)	0/574(0%)
		**ITS**	OM480726	*Kazachstania yasuniensis*	CLQCA 20–132	NR_160315	656/661(99%)	0/661(0%
*Limtongozyma* sp.	UFMG-CM-Y7105	**D1/D2**	OM321335	*Limtongozyma cylindracea*	CBS:6330	KY106404	478/489(98%)	0/489(0%)
*Lipomyces* sp. 1	UFMG-CM-Y7383	**D1/D2**	OR730887	*Lipomyces kononenkoae*	CBS 2514	AB614132	545/550(99%)	0/550(0%)
				*Lipomyces kockii*	CBS 7729	AB747652	545/550(99%)	0/550(0%)
				*Lipomyces mesembrius*	CBS 7661	AB747656	544/550(99%)	0/550(0%)
		**ITS**	OR754340	*Lipomyces spencermartinsiae*	CBS 5608	NR_138181	497/516(96%)	4/516(0%)
				*Lipomyces yarrowii*	CBS 7557	AB747663	498/517(96%)	5/517(0%)
*Lipomyces* sp. 2	UFMG-CM-Y7372	**D1/D2**	OR730904	*Lipomyces okinawensis*	NBRC 110,620	NG_060358	533/536(99%)	0/536(0%)
				*Lipomyces kononenkoae*	CBS 2514	AB747653	529/536(99%)	0/536(0%)
		**ITS**	OR754355	*Lipomyces okinawensis*	NBRC 110,620	NR_155341	490/493(99%)	0/493(0%)
				*Lipomyces yarrowii*	CBS 7557	JN943159.1	484/499(97%)	2/499(0%)
				*Lipomyces yamadae*	CBS 7532	NR_136955	483/498(97%)	1/498(0%)
*Lipomyces* sp. 3	UFMG-CM-Y7369	**D1/D2**	OR730876	*Lipomyces kononenkoae*	CBS 2514	KY108299	539/543(99%)	0/543(0%)
				*Lipomyces kockii*	CBS 7729	AB747652	537/543(99%)	0/543(0%)
				*Lipomyces starkeyi*	NRRL Y-11,557	JQ689072	537/543(99%)	0/543(0%)
		**ITS**	OR754329	*Lipomyces spencermartinsiae*	CBS 5608	NR_138181	511/518(99%)	2/518(0%)
				*Lipomyces kononenkoae*	CBS 2514	AB614132	511/518(99%)	2/518(0%)
*Lipomyces* sp. 4	UFMG-CM-Y7371	**D1/D2**	OR730897	*Lipomyces spencermartinsiae*	CBS 5608	AB747658	534/536(99%)	0/536(0%)
				*Lipomyces kononenkoae*	CBS 2514	AB747653	533/536(99%)	0/536(0%)
				*Lipomyces kockii*	CBS 7729	AB747652	531/536(99%)	0/536(0%)
		**ITS**	OR754350	*Lipomyces spencermartinsiae*	CBS 5608	NR_138181	504/508(99%)	3/508(0%)
				*Lipomyces kononenkoae*	CBS:2514	AB614132	504/508(99%)	3/508(0%)
*Lipomyces* sp. 5	UFMG-CM-Y7391	**D1/D2**	OR730898	*Lipomyces kononenkoae*	CBS 2514	AB747653	515/518(99%)	2/518(0%)
				*Lipomyces yamadae*	CBS 7532	AB747662	498/519(96%)	7/519(1%)
				*Lipomyces spencermartinsiae*	CBS 5608	DQ518980	497/519(96%)	6/519(1%)
		**ITS**	OR754351	*Lipomyces kononenkoae*	CBS 2514	NR_138180	515/518(99%)	2/518(0%)
				*Lipomyces yamadae*	CBS 7532	NR_136955	498/519(96%)	7/519(1%)
				*Lipomyces spencermartinsiae*	CBS 5608	AB747658	497/519(96%)	6/519(1%)
*Lipomyces* sp. 6	UFMG-CM-Y7377	**D1/D2**	OR730880	*Lipomyces kononenkoae*	CBS 2514	KY108299	561/562(99%)	0/562(0%)
				*Lipomyces kockii*	CBS 7729	AB747652	559/562(99%)	0/562(0%)
				*Lipomyces tetrasporus*	CBS 5910	NR_077108	559/562(99%)	0/562(0%)
		**ITS**	OR754332	*Lipomyces spencermartinsiae*	CBS 5608	NR_138181	513/528(97%)	1/528(0%)
				*Lipomyces kononenkoae*	CBS 2514	AB614132	513/528(97%)	1/528(0%)
				*Lipomyces yamadae*	CBS 7532	NR_136955	513/529(97%)	4/529(0%)
*Lipomyces* sp. 7	UFMG-CM-Y7370	**D1/D2**	OR730883	*Lipomyces kononenkoae*	CBS 2514	KY108299	547/548(99%)	0/548(0%)
				*Lipomyces kockii*	CBS 7729	AB747652	545/548(99%)	0/548(0%)
				*Lipomyces tetrasporus*	CBS 5910	NR_077108	545/548(99%)	0/548(0%)
		**ITS**	OR754335	*Lipomyces spencermartinsiae*	CBS 5608	NR_138181	482/496(97%)	1/496(0%)
				*Lipomyces kononenkoae*	CBS 2514	AB614132	482/496(97%)	1/496(0%)
				*Lipomyces yamadae*	CBS 7532	NR_136955	480/494(97%)	3/494(0%)
*Lipomyces* sp. 8	UFMG-CM-Y7375	**D1/D2**	OR730877	*Lipomyces orientalis*	CBS 10,300	PP809474	545/549(99%)	1/549(0%)
				*Lipomyces starkey*	NBRC 106,974	AB760325	545/548(99%)	0/548(0%)
		**ITS**	OR754330	*Lipomyces tetrasporus*	CBS 5910	NR_077108	490/504(97%)	4/504(0%)
*Metschnikowia* sp. 1	UFMG-CM-Y7124	**D1/D2**	OM321357	*Candida bromeliacearum*	UNESP 99 − 64	AY193780	315/347(91%)	14/347(4%)
*Metschnikowia* sp. 2	UFMG-CM-Y7163	**D1/D2**	OR211601	*Metschnikowia koreensis*	CBS 8854	NG_058340	398/484(82%)	6/484(1%)
*Metschnikowia* sp. 3	UFMG-CM-Y7107	**D1/D2**	OM321337	*Candida eppingiae*	CBS:8586	KY106433	301/341(88%)	16/341(4%)
*Nakaseomyces* sp.	UFMG-CM-Y7116	**D1/D2**	OM321347	*Candida nivariensis*	CBS 9983	MH545923	560/570(98%)	0/570(0%)
*Pichia* sp.	UFMG-CM-Y7123	**D1/D2**	OM321356	*Candida californica*	CBS:989	KY106378	534/560(95%)	0/560(0%)
*Schwanniomyces* sp.	UFMG-CM-Y7126	**D1/D2**	OM321359	*Schwanniomyces yamadae*	NRRL Y-11,714	NG_054862	488/538(91%)	6/538(1%)
*Sugiyamaella* sp.	UFMG-CM-Y7114	**D1/D2**	OM321345	*Sugiyamaella bonitensis*	UFMG-CM-Y608	KT006004	476/504(94%)	3/504(0%)
*Sungouiella* sp. 1	UFMG-CM-Y7120	**D1/D2**	OM321352	*Sungouiella inulinophila*	NBRC 106,735	AB550104	416/481(86%)	23/481(4%)
*Sungouiella* sp. 2	UFMG-CM-Y7165	**D1/D2**	OR426564	*Sungouiella middelhoveniana*	CBS 12,306	NG_060817	364/385(95%)	5/385(1%)
*Sungouiella* sp. 3	UFMG-CM-Y7161	**D1/D2**	OR211599	*Sungouiella pseudointermedia*	CBS 6918	MK394147	527/530(99%)	2/530(0%)
		**ITS**	OR228684	*Sungouiella pseudointermedia*	CBS 6918	MK394147	378/394(96%)	2/394(0%)
*Torulaspora* sp.	UFMG-CM-Y7122	**D1/D2**	OM321355	*Torulaspora franciscae*	CBS 2926	NG_055059	509/540(94%)	7/540(1%)
*Wickerhamiella* sp.	UFMG-CM-Y7121	**D1/D2**	OM321354	*Wickerhamiella musiphila*	CBS 10,697	NG_055366	457/492(93%)	4/492(0%)
*Wickerhamomyces* sp.	UFMG-CM-Y7099	**D1/D2**	OM321329	*Wickerhamomyces pijperi*	NBRC 102,059	AB449695	538/540(99%)	0/540(0%)
		**ITS**	OR228687	*Wickerhamomyces pijperi*	CBS:2887	KY105912	436/464(94%)	4/464(0%)
*Yamadazyma* sp. 1	UFMG-CM-Y6633	**D1/D2**	OM321326	*Candida vrieseae*	CBS 10,829	NG_060833	540/549(98%)	0/549(0%)
*Yamadazyma* sp. 2	UFMG-CM-Y7115	**D1/D2**	OM321346	*Candida songkhlaensis*	CBS:10,791	KY106765	503/510(99%)	0/510(0%)
*Yamadazyma* sp. 3	UFMG-CM-Y7117	**D1/D2**	OM321348	*Yamadazyma phyllophila*	DMKU RK548	NG_054772	468/475(99%)	0/475(0%)
*Papiliotrema* sp. 1	UFMG-CM-Y7166	**D1/D2**	OR211602	*Papiliotrema miconiae*	CBS 8358	AF444698	586/595(98%)	0/595(0%)
		**ITS**	OR228686	*Papiliotrema miconiae*	CBS 8358	AF444387	493/506(97%)	3/506(0%)
*Papiliotrema* sp. 2	UFMG-CM-Y7118	**D1/D2**	OM321350	*Papiliotrema laurentii*	CBS:139	KY108739	514/520(99%)	0/520(0%)
*Papiliotrema* sp. 3	UFMG-CM-Y7103	**D1/D2**	OM321333	*Papiliotrema siamensis*	DMKU SP85	NG_060062	542/547(99%)	0/547(0%)
		**ITS**	OM480727	*Papiliotrema perniciosus*	VKM Y-2905	NR_137653	475/481(99%)	1/481(0%)
*Papiliotrema* sp. 4	UFMG-CM-Y6631	**D1/D2**	OM321349	*Papiliotrema rajasthanensis*	CBS 10,406	NG_058366	529/535(99%)	0/535(0%)
		**ITS**	OM480723	*Papiliotrema rajasthanensis*	CBS 10,406	NR_155678	465/483(96%)	6/483(1%)
*Papiliotrema* sp. 5	UFMG-CM-Y7110	**D1/D2**	OM321341	*Papiliotrema mangalensis*	CBS:10,870	KY107130	523/533(98%)	0/533(0%)
*Tremella* sp.	UFMG-CM-Y7111	**D1/D2**	OM321342	*Tremella globispora*	CBS 6972	AF189869	510/524(97%)	1/524(0%)

Two novel *Geotrichum* species were represented by more than 10 isolates obtained from samples collected at “Piquiá,” TPI, and UFAM. The closest relatives of *Geotrichum* sp. 1 and *Geotrichum* sp. 2 are *G. geotrichum* and *G. europaeum*, respectively. These potential *Geotrichum* species differ by more than 10 nucleotide substitutions from their closest relatives in D1/D2 sequences. *Yamadazyma* sp. 1, represented by 12 isolates, was obtained from samples collected at “Piquiá.” *Yamadazyma* sp. 1 differs by nine nucleotides in D1/D2 sequences from *C. vrieseae*, its closest relative. This candidate for a novel species has a D1/D2 sequence identical to *Yamadazyma* sp. CLIB 1610, a yeast strain isolated in French Guiana. Eleven isolates, named *Candida* sp. 2—one from UFAM and ten from “Piquiá”—are related to *C. thaimueangensis*, a species belonging to the *Pichia* clade [68]. These species differ by 75 nucleotides and 19 indels in D1/D2 sequences. The isolation of this novel species only from soil in this biome suggests this substrate as a possible habitat for this yeast.

One noteworthy candidate for a novel species was *Candidozyma* sp., represented by a single isolate. This yeast was closely related to *Candidozyma ruelliae* and *C. haemulonii*. *Candidozyma* sp. 3 differs by 75 substitutions and 22 indels from *C. ruelliae* and by 76 substitutions and 19 indels from *C. haemulonii* in the D1/D2 sequences. *Candidozyma ruelliae* was described to accommodate two strains isolated from *Ruellia* sp. (Acanthaceae) flowers collected in India [69]. It was also found in soil samples from Zambia and air particles from Singapore [70]. *Candidozyma haemulonii* is a species complex formed by *C. haemulonii*, *C. haemulonii var. vulnera*, *C. duobushaemulonii*, *C. pseudohaemulonii*, and *C. vulturna*. These are clinically important species that can cause fungemia outbreaks and have a multidrug resistance profile [71]. The *C. haemulonii* species complex and *C. ruelliae* are phylogenetically related to the emerging multidrug-resistant yeast *Candidozyma auris* [72]. Species phylogenetically related to *C. auris* need further studies to determine their potential as opportunistic pathogens for humans. The isolation of yeast from this clade in Amazonian rainforest soils highlights the importance of monitoring preserved environments for the occurrence of yeasts with the potential to become emerging pathogens for humans. Other candidates for novel species occurred at low frequencies, mainly as singletons. It is necessary to increase collection efforts to isolate additional strains of these yeast species to determine their ecological niches in the Amazonian rainforest biome for further species description.

### Concluding remarks

To our knowledge, this is the first study comparing two isolation techniques and three different incubation temperatures for soil yeast isolation in tropical forest biomes. This is also the first study using selective medium for *Lipomyces* isolation from Brazilian Amazonian rainforest soils. Our results showed the occurrence of highly diverse yeast communities, especially in soils from *terra-firme* forest sites. Among the 110 yeast species found in this work, 45 are candidates for novel species, including most of the *Lipomyces* isolates. The use of different incubation temperatures, as well as different isolation techniques and/or media, can contribute to increasing the number of yeast species recovered in culture-dependent studies. Soil yeasts showed potential as a source of extracellular enzymes, especially xylanases, pectinases, esterases, and lipases, as well as phosphate solubilizers, which can be explored in further studies aimed at bioinnovation. This study demonstrates the potential of soils from the Brazilian Amazonian rainforest as a tremendous reservoir of novel yeast species.

## Data Availability

All data generated or analysed during this study are included in this published article [and its supplementary information files].
